# The Effect of Hemp (*Cannabis sativa* L.) Seeds and Hemp Seed Oil on Vascular Dysfunction in Obese Male Zucker Rats

**DOI:** 10.3390/nu13082575

**Published:** 2021-07-27

**Authors:** Michał Majewski, Adam Jurgoński

**Affiliations:** 1 Department of Pharmacology and Toxicology, Faculty of Medicine, UWM, 10-082 Olsztyn, Poland; 2 Division of Food Science, Institute of Animal Reproduction and Food Research, Polish Academy of Sciences, 10-748 Olsztyn, Poland

**Keywords:** glucose tolerance test, heart rate, mean arterial pressure, NS1619, pinacidil, thoracic aorta, thromboxane-A_2_, U-46619, Zucker rat

## Abstract

Seeds of industrial hemp (*Cannabis sativa* L.) contain a large amount of protein (26.3%), dietary fiber (27.5%), and fatty acids (33.2%), including linoleic, α-linolenic, and some amount of γ-linolenic acid. In our study, obese male Zucker rats (*n* = 6) at 8 weeks of age were supplemented for a further 4 weeks with either ground hemp seeds (12% diet) or lipid fractions in the form of hemp seed oil (4% diet). Hemp oil decreased blood plasma HDL-cholesterol (x0.76, *p* ≤ 0.0001), triglycerides (x0.55, *p* = 0.01), and calculated atherogenic parameters. Meanwhile, hemp seeds decreased HDL-cholesterol (x0.71, *p* ≤ 0.0001) and total cholesterol (x0.81, *p* = 0.006) but not the atherogenic index. The plasma antioxidant capacity of water-soluble compounds was decreased by the seeds (x0.30, *p* = 0.0015), which in turn was associated with a decrease in plasma uric acid (x0.18, *p* = 0.03). Dietary hemp seeds also decreased plasma urea (x0.80, *p* = 0.02), while the oil decreased the plasma total protein (x0.90, *p* = 0.05). Hemp seeds and the oil decreased lipid peroxidation in the blood plasma and in the heart (reflected as malondialdehyde content), improved contraction to noradrenaline, and up-regulated the sensitivity of potassium channels dependent on ATP and Ca^2+^. Meanwhile, acetylcholine-induced vasodilation was improved by hemp seeds exclusively. Dietary supplementation with ground hemp seeds was much more beneficial than the oil, which suggests that the lipid fractions are only partially responsible for this effect.

## 1. Introduction

Vascular dysfunction, including compromised vasodilation and vasoconstriction, is an important complication of chronic obesity [1]. Metabolic disorders associated with obesity, such as dyslipidemia, low-grade systemic inflammation, and increased oxidative stress, can lead to the development of atherosclerosis, hypertension, and some other cardiovascular disorders [2]. Therefore, there is a need to find ways to prevent and treat the early development of obesity with its complications. A good experimental model of obesity and vascular dysfunction is homozygous recessive Zucker rats (*fa*/*fa*), which have a mutation in a gene-encoding leptin receptor, resulting in a lack of sensitivity to circulating leptin in the blood [1,3]. In Zucker rats, leptin is unable to inhibit neuropeptide Y secretion in the hypothalamus that potentiates appetite, and thus a meaningful increase in dietary intake and body weight is observed together with the occurrence of the aforementioned metabolic disorders [3].

An increased contribution of plant-based food is one of the ways to prevent obesity and associated metabolic disorders, which is partly due to the relatively low caloric value of such food [4]. However, food from plants is also a good source of bioactive compounds that can directly bring benefits to the cardiovascular system, a good example of which are polyunsaturated fatty acids (PUFAs) with α-linolenic acid and linoleic acid as their main representatives in nature [5]. Besides their nutritional importance, these PUFAs, especially from the n-3 family, can improve lipid metabolism and the inhibition of lipid synthesis in the body [6,7]. PUFAs are also believed to prevent lipid accumulation in the arteries and decrease the development of hypertension. However, the consumption of PUFAs can also bring some negative consequences to the body, which especially applies to the fatty acids of the n-6 family that can be metabolized to pro-inflammatory eicosanoids [7].

An interesting and relatively less known source of fatty acids is the seeds of industrial hemp (*Cannabis sativa* L.), as they contain a large amount of PUFAs, including linoleic and α-linolenic acids (approx. 53% and 18% of total fatty acids) and some small amount of other fatty acids that are uncommon in vegetable oils, such as γ-linolenic acid [8,9]. The oil from hemp seeds is characterized as having an optimal ratio of n-3 to n-6 PUFAs (1:3), so important for the adequate functioning of the cardiovascular system; however, hemp oil contains trace amounts of trans fatty acids, which are generally thought to raise the risk of atherosclerosis by inhibiting the synthesis of other PUFAs in arterial cells [7,9]. Nevertheless, Al-Khalifa et al. [10] demonstrated that hearts from rats fed with hemp seeds exhibited significantly better postischemic recovery of maximal contractile function and relaxation during reperfusion compared to the control group. The authors concluded that this was due to PUFAs from hemp seeds. However, hemp seeds are also rich in peptides that have been recognized as a potential antihypertensive agent [11] and lignan amides with potential anti-inflammatory and cardiovascular activities [12]. Moreover, in the latest study conducted in one of our laboratories, relatively strong hypolipidemic effects of dietary hemp in genetically obese rats were observed, and the oil fraction was only partially responsible for these effects [9].

The aforementioned findings suggest that the consumption of hemp seeds is beneficial for the cardiovascular system compromised by obesity. However, the extent to which PUFAs derived from hemp seeds can be responsible for these beneficial effects has not yet been studied nor the mechanisms underlying their vascular protection. Thus, we aimed to compare the effects of hemp seeds (12% diet) with the corresponding amount of lipid fractions from hemp seed oil (4% diet) on the cardio-vascular system in genetically obese Zucker rats. We hypothesized that supplementation with either the seeds or the oil can improve metabolic dysfunction of genetic origin and that the seeds are more effective due to a wider range of potentially bioactive compounds.

## 2. Materials and Methods

### 2.1. Drugs and Chemicals

Acetylcholine chloride, sodium nitroprusside, and noradrenaline hydrochloride were obtained from Sigma-Aldrich (St. Louise, MO, USA); potassium chloride from Chempur (Piekary Śląskie, Poland); pinacidil, NS1619, and U-46619 from Cayman Chemical (Ann Arbor, MI, USA). Stock solutions (10 mM) of these drugs were prepared in distilled water, except for noradrenaline, which was dissolved in NaCl (0.9%) + ascorbic acid (0.01% *w*/*v*) solution; pinacidil, NS-1619 were dissolved in DMSO, and U-46619 in ethanol.

These solutions were stored at −20 °C, and appropriate dilutions were made in Krebs-Henseleit solution (KH in mmol/L: NaCl 115; CaCl_2_ 2.5; KCl 4.6; KH_2_PO_4_ 1.2; MgSO_4_ 1.2; NaHCO_3_ 25; glucose 11.1) on the day of the experiment. The maximal solvent concentration in the medium was less than 0.01% (*vol*/*vol*). At these concentrations, solvents did not alter the reactivity of the studied arteries. 

Hemp seeds were purchased from Ekogram (Zielonki, Poland), and unrefined, cold-pressed hemp seed oil was obtained from Ol’Vita (Panków, Poland).

### 2.2. Chemical Composition of Hemp Seeds and Hemp Seed Oil

Following methods were applied: gravimetric method at a high temperature 105 °C/∼580 °C for dry matter (DM) content and ash; enzymatic–gravimetric method for total dietary fiber; Kjeldahl method for crude protein; the Soxhlet extraction for crude fat; and gas chromatography with flame ionization detection (Hewlett Packard 5890, Wilmington, DE, USA) for fatty acids methyl esters, as previously described [5].

### 2.3. Experimental Protocol

All efforts were made to minimize animal suffering. Male Zucker rats (Charles River, Sulzfeld, Germany) at 8 weeks of age were randomly allocated to 4 groups (*n* = 6/group) and fed for a further 4 weeks with experimental diets in form of pellets. The lean controls (LC) and obese controls (OC) were fed with a standard rat chow, whereas the other 2 obese groups were fed a modified diet in which either hemp seeds (12% diet; HS) or hemp seed oil (4% diet; HO) were included. All these diets were prepared in such a manner so that they had the same amount of carbohydrate (52%), protein (18%), fat (8.3%), and fiber (5%). Diets fed to the O+HS group and O+HO group also had a similar fatty acid profile.

The rats were individually housed in plastic cages under a controlled environment (a 12 h light–dark cycle, a temperature of 21 ± 1 °C, relative humidity of 50–70%, and 20 air changes per hour) [13].

### 2.4. Experimental Procedures

The mixture of ketamine + xylazine (100 + 10 mg/kg BW) was used for intraperitoneal anesthesia [14]. The whole blood was kept in tubes containing heparin + EDTA as an anticoagulant and centrifuged at 3000× *g* for 10 min to separate the blood plasma, which was stored at –80 °C until further analysis. The thoracic arteries were carefully isolated and kept in a Krebs–Henseleit buffer at +4 °C. 

### 2.5. Blood and Heart Tissue Analysis

The traditional blood plasma lipids (the total cholesterol—TC, high-density lipoprotein cholesterol—HDL, triglycerides—TG; mmol/L), gamma-glutamyl transferase (GGT, U/L), uric acid (µmol/L), urea (mmol/L), creatinine level (µmol/L), albumin (µmol/L), and total protein (g/L) were measured with a biochemical autoanalyzer (Horiba, Kyoto, Japan) [14].

The malondialdehyde-thiobarbituric acid (MDA-TBA) adduct was quantified at 532/553 nm (Ex/Em) with a Fluorometric Assay Kit (ab118970), and the values were expressed as µmol/L of the blood plasma and ng/g of heart tissue. 

The antioxidant capacity of water- and lipid-soluble compounds of the blood plasma (ACW and ACL, respectively; mg/L) were determined by a photo-chemiluminescence detection method using Photochem (Analytik Jena AG, Germany). This method is based on the generation of free radicals that are partially eliminated through a chemical reaction with antioxidants present in the plasma sample, and the remaining radicals are quantified by luminescence generation. The lipid fraction was separated using methanol, n-hexane, and centrifugation. Calibration curves are based on Ascorbate and Trolox as standards for ACW and ACL, respectively.

### 2.6. Glucose Tolerance Test

Rats were given a 50% glucose solution by oral gavage (2 g/kg of body weight) after overnight starvation, 4 days before the final termination. Blood samples were collected from the tail tip, and glucose was measured with a glucometer (Accu-Chek Active, Roche Diagnostics, Germany) at 0, 15, 30, 60, 90, 120, and 180 min. 

### 2.7. Blood Pressure Measurements

Mean arterial pressure, MAP (mmHg) and heart rate, HR (bpm) were monitored on the day before the blood collection with the noninvasive tail-cuff method (LE5001, Panlab, Harvard Apparatus, Barcelona, Spain).

### 2.8. Thromboxane-A_2_ Quantification

This was completed following Majewski et al. [2]. Briefly, after a stabilization period in KHS at +37 °C for 30 min (pH 7.4), aortic rings from each group of rats were followed by 2 wash periods of 10 min using 200 μL of KHS. Once fresh KHS was replaced, arteries were exposed to noradrenaline (0.1 μM, 2 min) and then to the cumulative acetylcholine concentrations (0.1 nM–10 μM) at 1 min intervals. The medium was collected and stored at −80 °C until further analysis. Production of thromboxane-A_2_ was monitored by measuring the stable metabolite thromboxane-B_2_. This was completed using the appropriate enzyme immunoassay kit (Cayman Chemical, Ann Arbor, MI, USA). Results are expressed as pg/mg of tissue.

### 2.9. Vascular Reactivity Studies

This was previously described in detail by Majewski et al. [14,15]. Briefly, isolated aortic rings of 4 mm length were aerated in a stagnant 5 mL Graz Tissue Bath System (Harvard Apparatus, March-Hugstetten, Germany) for 60 min, under 1 g pre-load tension, and precontracted with noradrenaline (0.1 μM). Then, the cumulative concentrations of either acetylcholine (0.1 nM–10 μM), sodium nitroprusside (0.001 nM–10 μM), pinacidil (10 nM–10 μM), or NS-1619 (1 nM–10 μM) were added into the bath chambers to study the vasodilator response. In another set of experiments, vasoconstriction was studied with cumulative concentrations of noradrenaline (0.1 nM–10 μM) and U-46619 (0.1 nM–1 μM). 

### 2.10. Data Analysis and Statistics 

A nontraditional lipid profile was calculated based on TC, HDL, and TG as log_10_$\left(\frac{\mathrm{TG}}{\mathrm{HDL}}\right)$, $\frac{\mathrm{TC}}{\mathrm{HDL}}$, TC _minus_ HDL, VLDL (calculated as: $\frac{\mathrm{TG}}{2.2}$), LDL (calculated as TC _minus_ HDL _minus_ VLDL), $\frac{\mathrm{TG}*\mathrm{TC}*\mathrm{LDL}}{\mathrm{HDL}}$, $\frac{\mathrm{nonHDL}}{\mathrm{HDL}}$ and $\frac{\mathrm{LDL}}{\mathrm{HDL}}$. _Non_HDL was calculated as TC _minus_ HDL [14,16,17]. MAP was calculated as DP + 1/3(SP − DP), where DP is the diastolic blood pressure and SP is the systolic blood pressure.

The contraction induced by high KCl (75 mM) was expressed in mg of developed tension; meanwhile, contraction with noradrenaline and U-46619 was expressed as % of KCl-induced response. Vascular relaxation was expressed as a percentage of the contractile response to noradrenaline NA (0.1 μM). This concentration of NA was chosen based on the preliminary studies with cumulative doses of NA added into the incubation chambers. The cumulative concentration–response curves were analyzed by a nonlinear regression model, which determined the area under the curve (AUC), maximal response (E_max_, %), and the potency (pEC_50_). The group comparison was performed by either a parametric (ANOVA) or non-parametric test (Kruskal–Wallis test), with *n* = 6. The Gaussian distribution of residuals and homoscedasticity of variance were tested. The Grubbs’ test was performed to detect outliers. The post hoc tests were run only when F achieved the necessary level of statistical significance (*p* ≤ 0.05). The group comparison was performed by Mann–Whitney’s test. Results are expressed as means ± SD (and means ± SEM for vascular studies). This research was randomized and stayed blinded for laboratory analyses. The level of significance was when *p* ≤ 0.05.

## 3. Results

The composition of hemp seeds and hemp seed oil was determined in order to prepare the experimental diets. Crude fat was calculated as 100:33.2, so the concentration of hemp seeds was increased three-fold compared with the oil; see Table 1. 

### 3.1. The General Characterization of Experimental Animals

**Figure 1:**
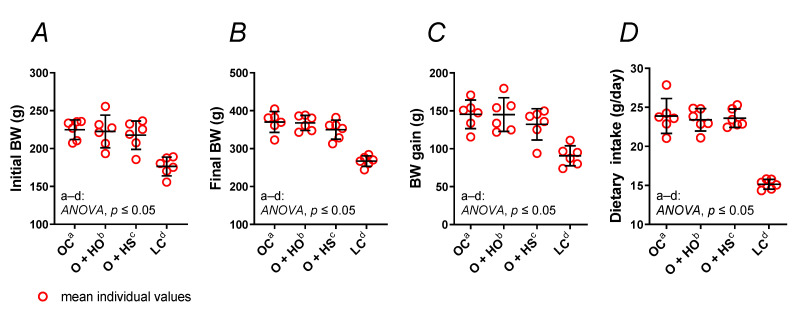
Body weight (BW) (**A**–**C**) and dietary intake (**D**) of 8-week-old lean (LC) and obese (OC) Zucker rats fed a diet containing hemp seed oil (HO) and hemp seeds (HS) for 4 weeks. Values are means ± SD, *n* = 6, *p* ≤ 0.05 (two-way ANOVA). Experimental supplementation with HO and HS neither changed the body weight nor the dietary intake of obese Zucker rats.

Experimental supplementation with HO neither changed the body weight (Figure 1A–C) nor the dietary intake (Figure 1D) of obese Zucker rats (x1.00, *p >* 0.9999, and x0.98, *p* = 0.94, respectively), and the same was found for the HS-group of rats (x0.91, *p* = 0.66, and x0.99, *p* = 0.99, respectively). No significant difference was observed in HS- vs. HO-supplemented rats (x0.91, *p* = 0.68, and x1.01, *p* = 0.99, respectively); see Table S1.

### 3.2. The Lipid Profile

**Figure 2:**
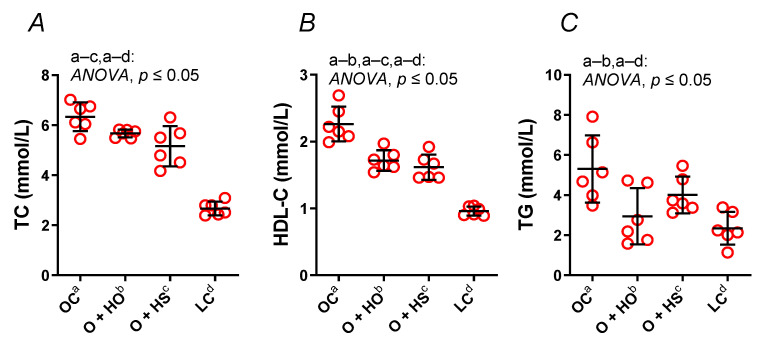
Total cholesterol (TC) (**A**), high-density lipoprotein cholesterol (HDL-C) (**B**), and triglycerides (TG) (**C**) in blood plasma of 8-week-old lean (LC) and obese (OC) Zucker rats fed a diet containing hemp seed oil (HO) and hemp seeds (HS) for a further 4 weeks. Values are means ± SD, *n* = 6, *p* ≤ 0.05 (two-way ANOVA). HO decreased the HDL and TG; meanwhile, HS decreased the HDL and TC.

Supplementation with HO significantly decreased both the HDL (x0.76, *p* ≤ 0.0001) and TG (x0.55, *p* = 0.01), but not the TC (x0.89, *p* = 0.16). In contrast, HS decreased the TC (x0.81, *p* = 0.006) and the HDL (x0.71, *p* ≤ 0.0001) but not the TG (x0.76, *p* = 0.25); see Figure 2A–C. No significant difference in the lipid profile was observed in HS- vs. HO-supplemented rats (TC x0.91, *p* = 0.35; HDL x0.94, *p* = 0.6; TG x1.36, *p* = 0.4).

### 3.3. The Calculated Nontraditional Lipid Profile

**Figure 3:**
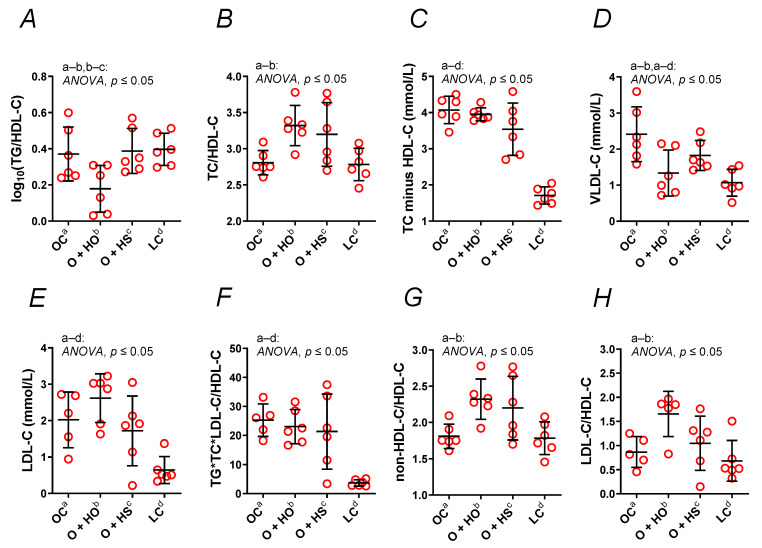
A nontraditional lipid profile (**A**–**H**) of 8-week-old lean (LC) and obese (OC) Zucker rats fed a diet containing hemp seed oil (HO) and hemp seeds (HS) for 4 weeks. Values are means ± SD, *n* = 6, *p* ≤ 0.05 (two-way ANOVA). HO decreased the calculated atherogenic parameters; meanwhile, HS had no such effect.

TC minus HDL, LDL, $\frac{\mathrm{TG}*\mathrm{TC}*\mathrm{LDL}}{\mathrm{HDL}}$ were not modified by the hemp opposite to the log_10_$\left(\frac{\mathrm{TG}}{\mathrm{HDL}}\right)$, $\frac{\mathrm{TC}}{\mathrm{HDL}}$, VLDL, $\frac{\mathrm{nonHDL}}{\mathrm{HDL}}$, and $\frac{\mathrm{LDL}}{\mathrm{HDL}}$ (Figure 3A–H). The HO decreased VLDL (x0.55, *p* = 0.013) and log_10_$\left(\frac{\mathrm{TG}}{\mathrm{HDL}}\right)$ (x0.48, *p* = 0.023); meanwhile, it increased $\frac{\mathrm{LDL}}{\mathrm{HDL}}$ (x1.80, *p* = 0.04),$\;\frac{\mathrm{nonHDL}}{\mathrm{HDL}}$ (x1.28, *p* = 0.016), and $\frac{\mathrm{TC}}{\mathrm{HDL}}$ (x1.18, *p* = 0.016). The LDL (x1.29, *p* = 0.46), TC minus HDL (x0.97, *p* = 0.96), and $\frac{\mathrm{TG}*\mathrm{TC}*\mathrm{LDL}}{\mathrm{HDL}}$ (x0.91, *p* = 0.94) were not modified in a significant way. In contrast, HS did not have any significant effect on the calculated lipid profile ($\frac{\mathrm{nonHDL}}{\mathrm{HDL}}$ x1.22, *p* = 0.08; $\frac{\mathrm{LDL}}{\mathrm{HDL}}$ x1.21, *p* = 0.91; $\frac{\mathrm{TC}}{\mathrm{HDL}}$ x1.14, *p* = 0.08; log_10_$\left(\frac{\mathrm{TG}}{\mathrm{HDL}}\right)$ x1.05, *p* = 0.99; TC minus HDL x0.87, *p* = 0.20; LDL x0.85, *p* = 0.84; $\frac{\mathrm{TG}*\mathrm{TC}*\mathrm{LDL}}{\mathrm{HDL}}$ x0.85, *p* = 0.79; VLDL x0.76, *p* = 0.25). A significant increase in log_10_$\left(\frac{\mathrm{TG}}{\mathrm{HDL}}\right)$ (x2.17, *p* = 0.01) was observed in HS- vs. HO-supplemented rats. This was not observed for VLDL (x1.36, *p* = 0.40), $\frac{\mathrm{TC}}{\mathrm{HDL}}$ (x0.96, *p* = 0.84), $\frac{\mathrm{nonHDL}}{\mathrm{HDL}}$ (x0.95, *p* = 0.84), $\frac{\mathrm{TG}*\mathrm{TC}*\mathrm{LDL}}{\mathrm{HDL}}$ (x0.93, *p* = 0.98), TC minus HDL (x0.90, *p* = 0.40), $\frac{\mathrm{LDL}}{\mathrm{HDL}}$ (x0.67, *p* = 0.17), or LDL (x0.66, *p* = 0.10); see Table S1.

### 3.4. Markers of Antioxidant Status

**Figure 4:**
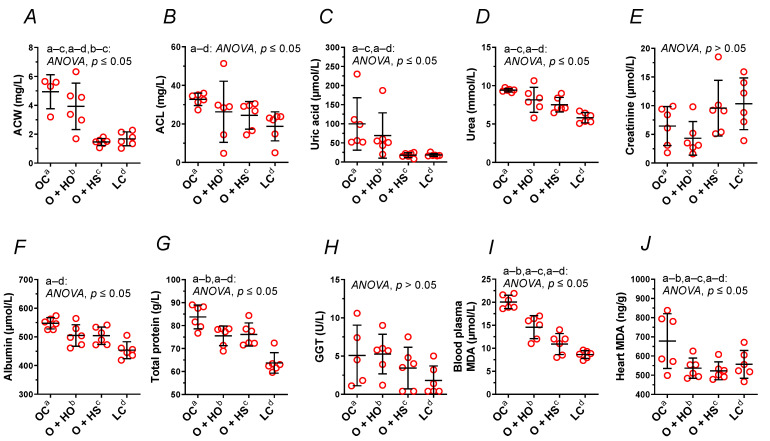
Blood plasma analysis (**A**–**I**) and heart malondialdehyde (**J**) of 8-week-old lean (LC) and obese (OC) Zucker rats fed a diet containing hemp seed oil (HO) and hemp seeds (HS) for 4 weeks. Values are means ± SD, *n* = 6, *p* ≤ 0.05 (two-way ANOVA). ACW is a marker that distinguishes HO from HS. ACL, antioxidant capacity of lipid-soluble compounds; ACW, antioxidant capacity of water-soluble compounds; MDA, malondialdehyde.

Supplementation with HO neither modified the ACW (x0.80, *p* = 0.63) nor the ACL (x0.80, *p* = 0.45); meanwhile, HS decreased ACW (x0.30, *p* = 0.0015) but not the ACL (x0.75, *p* = 0.25). A significant decrease in ACW (x0.37, *p* = 0.0048) was observed in HS- vs. HO-supplemented rats. This was not observed for the ACL (x0.93, *p* = 0.97); see Figure 4A,B and Table S1.

### 3.5. Blood Analysis

Supplementation with HO decreased the total protein (x0.90, *p* = 0.05), but not the GGT (x1.04, *p* = 1.00), albumin (x0.92, *p* = 0.08), urea (x0.87, *p* = 0.15), uric acid (x0.69, *p* = 0.66), and creatinine (x0.67, *p* = 0.84). By contrast, HS decreased the uric acid (x0.18, *p* = 0.03) and the urea (x0.80, *p* = 0.02). Neither the creatinine (x1.48, *p* = 0.63), the albumin (x0.92, *p* = 0.08), the total protein (x0.91, *p* = 0.07), nor GGT (x0.68, *p* = 0.86) were modified in a significant way. There was no significant difference between HS vs. HO in the level of creatinine (x2.22, *p* = 0.21), total protein (x1.01, *p* = 1.00), albumin (x1.00, *p >* 0.9999), urea (x0.92, *p* = 0.67), GGT (x0.65, *p* = 0.71), or uric acid (x0.26, *p* = 0.25); see Figure 4C–H and Table S1.

### 3.6. Lipid Peroxidation in Blood Plasma and Heart

Measurement of MDA showed increased lipid peroxidation in OC vs. LC. Supplementation with either HO or HS decreased MDA in blood plasma (x0.73, *p* = 0.0009 and x0.54, *p* < 0.0001, respectively, Figure 4I) and in the heart (x0.79, *p* = 0.05 and x0.77, *p* = 0.0441, respectively, Figure 4J). There was a significant difference between HS and HO in blood plasma MDA (x0.75, *p* = 0.0241) but not in the heart (x0.97, *p* = 0.6336).

### 3.7. Oral Glucose Tolerance Test (OGTT)

**Figure 5:**
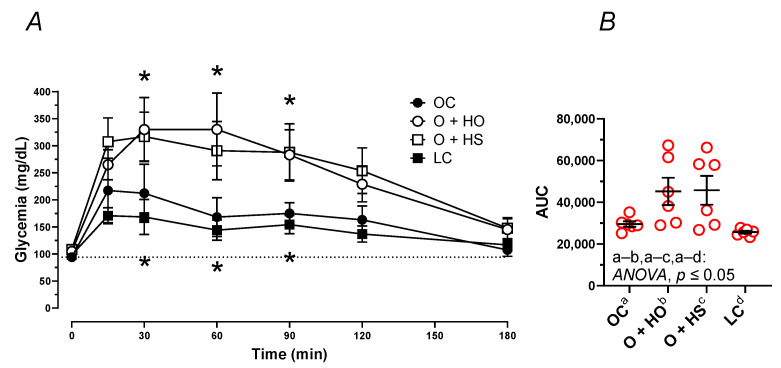
Oral glucose tolerance test (OGTT) of 8-week-old lean (LC) and obese (OC) Zucker rats fed a diet containing hemp seed oil (HO) and hemp seeds (HS) for 4 weeks. Values are means ± SD, *n* = 6, * *p* ≤ 0.05 vs. OC (two-way ANOVA) at 15, 30, 60, 90, 120, and 180 min (**A**), and as area under the curve—AUC (**B**). HO and HS did not ameliorate the impaired glucose tolerance test.

The OGTT pointed to the elevation of the blood glucose concentration in OC vs. LC (AUC: x1.15, *p* = 0.0252) after the administration of glucose (Figure 5A,B). Supplementation with HO and HS further elevated the blood plasma glucose (AUC: x1.53, *p* = 0.05 and x1.55, *p* = 0.05, respectively).

Blood plasma fasting glucose only tended to be different among the dietary groups *p* ≥ 0.1054, with the highest level observed in HS; see Table S1. 

### 3.8. Blood Pressure Measurements

**Figure 6:**
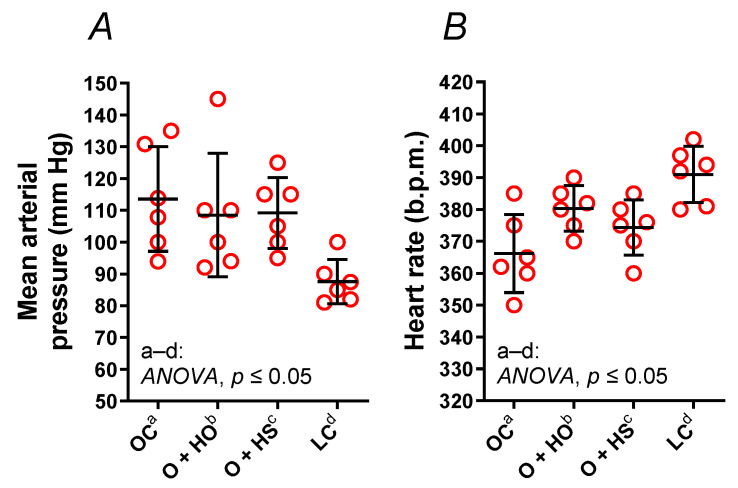
Mean arterial pressure (**A**) and a heart rate (**B**) of 8-week-old lean (LC) and obese (OC) Zucker rats fed a diet containing hemp seed oil (HO) and hemp seeds (HS) for 4 weeks. Values are means ± SD, *n* = 6, *p* ≤ 0.05 (two-way ANOVA). HO and HS had no impact on mean arterial pressure and heart rate.

The mean arterial pressure (MAP, x1.30, *p* = 0.0244, Figure 6A) and heart rate (HR, x0.94, *p* = 0.0010, Figure 6B) were modified in OC vs. LC. Experimental supplementation had no influence on MAP (HO: x0.96, *p* = 0.9501 and HS: x0.96, *p* = 0.6364) nor on HR (HO: x1.04, *p* = 0.0728 and HS: x1.02, *p* = 0.4529).

### 3.9. Thromboxane-A_2_ Quantification

**Figure 7:**
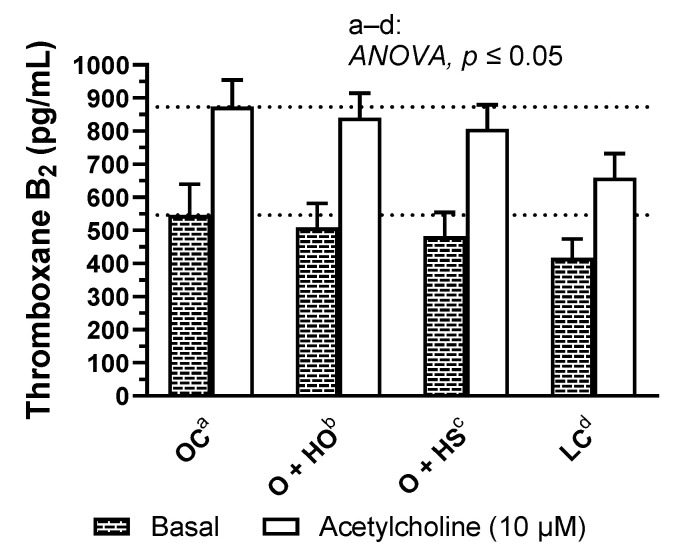
Basal- and acetylcholine-induced TXA_2_ release from thoracic arteries of 8-week-old lean (LC) and obese (OC) Zucker rats fed a diet containing hemp seed oil (HO) and hemp seeds (HS) for 4 weeks. Values are means ± SD, *n* = 6, *p* ≤ 0.05 (two-way ANOVA). HO and HS had no influence on basal and acetylcholine-induced TxA_2_ release in arteries.

Supplementation with hemp had no significant influence on basal and acetylcholine induced TxA_2_ release in rat arteries (HO: x0.93, *p* = 0.82 and x0.96, *p* = 0.87; HS: x0.88, *p* = 0.45 and x0.92, *p* = 0.43, HS vs. HO, x0.95, *p* = 0.93 and x0.96, *p* = 0.87, respectively); see Figure 7 and Table S1.

### 3.10. Vascular Reactivity Studies

**Figure 8:**
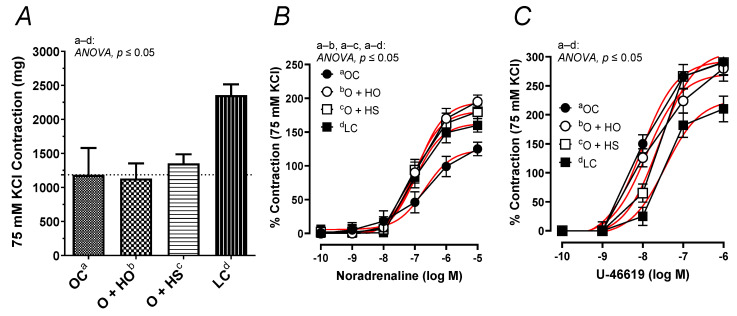
The contractile response of isolated thoracic arteries from 8-week-old lean (LC) and obese (OC) Zucker rats fed a diet containing hemp seed oil (HO) and hemp seeds (HS) for 4 weeks. Vascular contraction was induced by potassium chloride (75 mM KCl) (**A**), noradrenaline (0.1 nM–10 μM) (**B**), and a thromboxane-A_2_ analog (U-46619, 0.1 nM–1 μM) (**C**). Results (means ± SEM) are expressed in mg of tension and as a percentage of the previous tone elicited by 75 mM KCl. *p* ≤ 0.05 (two-way ANOVA/Sidak’s), *n* = 6. HO and HS improved contraction to noradrenaline.

The contractile response to 75 mM KCl was neither modified by the HO (x0.95, *p* = 0.33) nor by the HS (x1.14, *p* = 0.71). No significant difference in the KCl-induced contraction was observed in HS- vs. HO-supplemented rats (x1.20, *p* = 0.67); see Figure 8A. In contrast, the noradrenaline-induced contraction was increased in the group of rats supplemented with HO (ΔAUC, x1.59, *p* = 0.01) and HS (ΔAUC, x1.54, *p* = 0.03), but not in HS vs. HO (ΔAUC, x0.97, *p* = 0.9); see Figure 8B. Supplementation with hemp had no significant influence on the vascular contraction induced by U-46619 in obese Zucker rats (ΔAUC, HO x0.87, *p* = 0.4; HS x0.85, *p* = 0.5; HS vs. HO x0.97, *p* = 0.9); see Figure 8C.

**Figure 9:**
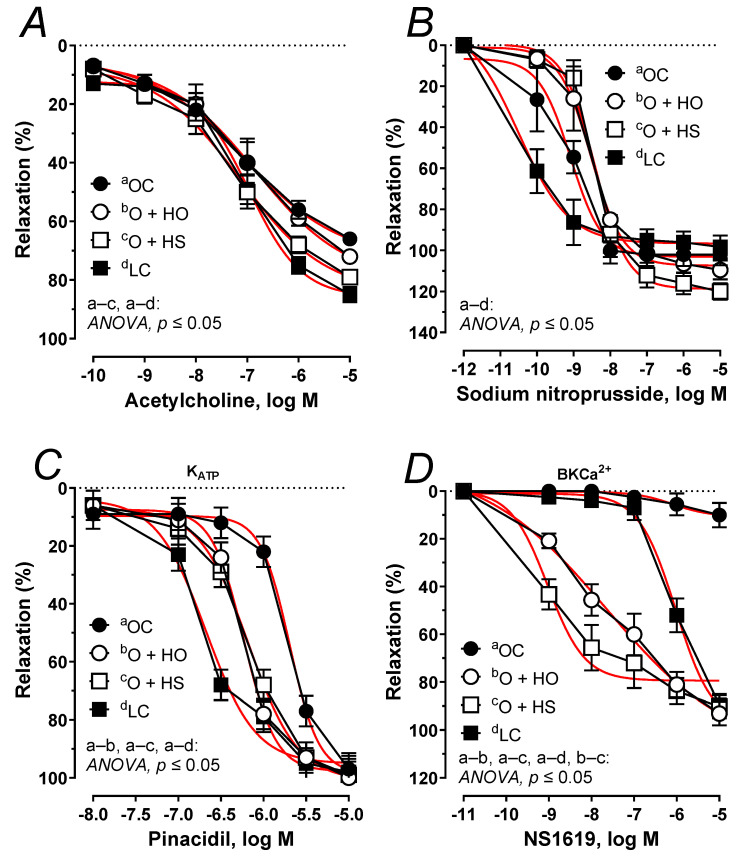
The relaxant response to acetylcholine (**A**), sodium nitroprusside (**B**), the K_ATP_ channel opener, pinacidil (**C**), and the BKCa^2+^ channel opener, NS1619 (**D**) of isolated thoracic arteries from 8-week-old lean (LC) and obese (OC) Zucker rats fed a diet containing hemp seed oil (HO) and hemp seeds (HS) for 4 weeks. Results (means ± SEM) are expressed as a percentage of the inhibition of the contraction induced by noradrenaline (0.1 μM), *n* = 6, *p* ≤ 0.05 (two-way ANOVA/Sidak’s). HO and HS improved functioning of potassium channels dependent on ATP and Ca^2+^. Moreover, HS improved the impaired vascular relaxation to acetylcholine.

The acetylcholine-induced relaxation was potentiated 1.21-fold by the HS (ΔAUC, *p* ≤ 0.05), but not by the HO (ΔAUC, x1.02, *p* = 0.45), Figure 9A. No significant difference was observed in HS vs. HO (ΔAUC, x1.19, *p >* 0.9). In both studied groups, the arteries precontracted with noradrenaline responded in a similar way when subjected to sodium nitroprusside (ΔAUC, HO, x0.86, *p >* 0.9; HS, x0.91, *p >* 0.5; and HS vs. HO x1.06, *p >* 0.9), Figure 9B. Relaxant response to pinacidil was shifted to the right x1.47 by the HO (ΔAUC, *p* ≤ 0.01), and x1.46 by the HS (ΔAUC, *p* ≤ 0.01); however, there was no significant difference in HS- vs. HO-induced relaxation (ΔAUC, x1.00, *p* = 0.97), Figure 9C. Relaxant response to NS1619 was increased x69.60 by the HO (ΔAUC, *p* ≤ 0.001), and x82.29 by HS (ΔAUC, *p* ≤ 0.001), and a significant change was observed in HS vs. HO (ΔAUC, x1.18, *p* ≤ 0.05); Figure 9D. For the E_max_, pEC_50_, and AUC, see Table S2.

## 4. Discussion

Previously, we had reported that ground seeds from dietary hemp (*Cannabis sativa* L.) more effectively attenuate metabolic disorders compared with the oil fraction from hemp seeds [9]. Now, we have further investigated the influence of dietary supplementation with hemp seeds (12% of diet) vs. corresponding concentration of hemp seed oil (4% of diet) on vascular dysfunction, blood pressure, and heart rate, the blood plasma lipid profile, oral glucose tolerance, antioxidant capacity, and renal functioning in obese Zucker rats, a model of obesity.

As was stated before, experimental supplementation neither modified the body weight gain nor the food intake of supplemented obese Zucker rats [9].

The influence of the oil from hemp seeds on lipid metabolism is well documented, contrary to the effect of the ground seeds, which is poorly studied as of yet. Therefore, we have undertaken further research. Both ground seeds and the oil were able to affect the lipid metabolism (decrease in the plasma HDL cholesterol), although the effectiveness of the seeds was much more indicated (decrease in the plasma total cholesterol). The plasma triglycerides concentration was not significantly decreased by the seeds, but it was by the oil, which is in accordance with our previous results [9]. We have further calculated a nontraditional lipid profile, which is an even better marker of atherogenicity [14,16,18]. Surprisingly, the effect of hemp oil was much more pronounced compared to the seeds, as indicated by a decrease in the AIP: log_10_$\left(\frac{\mathrm{TG}}{\mathrm{HDL}}\right)$, the cholesterol ratio: $\frac{\mathrm{TC}}{\mathrm{HDL}}$, VLDL, and by an increase in $\frac{\mathrm{nonHDL}}{\mathrm{HDL}}$ and $\frac{\mathrm{LDL}}{\mathrm{HDL}}$. Neither hemp seeds nor hemp oil improved the impaired glucose tolerance that was induced in the obese group of rats, yet these favorable effects of hemp seeds and oil were not as defined on blood glucose as those on the lipids. 

Hemp seeds are a good source of fatty acids (33.2%), see Table 1. In our study, the fatty acid profile of the seeds and the oil from hemp was found to be similar. The main PUFAs determined were linoleic acid, α-linolenic acid, and γ-linolenic acid, with an abundance of ∼52%, 18%, and 4%. Moreover, hemp seeds are also a good source of protein (26.3%) and dietary fiber (27.5%), which may explain the observed differences in the blood plasma lipids. Highly digestible proteins and dietary fiber can trigger a rise in protein synthesis of smooth muscles [19] and increase the gut microbial glycolytic activity: β-glucosidase as well as α- and β-galactosidase [9].

In addition, this study is the first to describe the reactivity of isolated thoracic arteries in hemp-supplemented obese Zucker rats. This specific rat model is characterized by a number of metabolic disorders, including vascular dysfunction and increased oxidative stress. Supplementation with hemp seeds and seed oil beneficially potentiated (already decreased, Figure 8B) vasoconstriction in response to noradrenaline. However, this neither changed the membrane depolarization induced by high KCl nor the response to the thromboxane-A_2_ analog, U-46619. It is worth mentioning that metabolic dysfunction observed in obese Zucker rats decreased depolarization and enhanced U-46619-induced contraction, as was presented in Figure 8A,C. Moreover, supplementation with hemp did not decrease the thromboxane-A_2_ level in blood vessels under basal and acetylcholine-stimulated conditions in obese Zucker rats.

Next, we studied impaired vascular relaxation observed in obese Zucker rats. The attenuated relaxant response to acetylcholine (Figure 9A) was improved by the seeds but not by the oil. Surprisingly, the vasodilator response to exogenous nitric oxide (which is attenuated in obese Zucker rats (Figure 9B) was not modified with dietary hemp. This indicates that the sensitivity of the smooth muscles of rat thoracic aorta to nitric oxide was not modified during supplementation and that it was the endothelial functioning that was improved by the seeds but not by the oil. In rat thoracic arteries, K_ATP_ and BK_Ca_ channels are also engaged in vascular tone regulation to compensate for the attenuated vascular relaxation [2]. The relaxant response to the K_ATP_ channel opener was down-regulated in obese rats, which points to a decreased sensitivity (Figure 9C). Experimental supplementation with hemp improved the impaired functioning of these channels and shifted that response to the left. However, the sensitivity was not fully restored to the level observed in the lean controls. Next, we examined the impaired vasodilator response with a BK_Ca_ channel opener (Figure 9D). In our study, supplementation with hemp increased both the sensitivity and the maximal response, which was more pronounced in rats fed with seeds (increased sensitivity). Our results point to an improvement in the functioning of K_ATP_ and BK_Ca_ channels in response to the dietary hemp, with a more beneficial effect from the seeds than the oil.

Despite these beneficial effects on the vascular system, neither HO nor HS had any beneficial impact on impaired mean arterial pressure and heart rate of obese Zucker rats.

We noticed an increased plasma antioxidant capacity of lipid- and water-soluble compounds (Figure 4A,B) as well as MDA in the blood plasma and the heart (Figure 4I,J) in obese Zucker rats compared to the lean controls, perhaps as a response to the increased oxidative stress, which up-regulated the mechanism(s) responsible for the antioxidant defense and potentiated lipid peroxidation. Both dietary seeds and the oil decreased the lipid peroxidation in the blood plasma and in the heart. However, the effectiveness of the seeds was more indicated, which was reflected by a decrease in the plasma antioxidant capacity of water-soluble compounds. This was not observed for hemp oil and the plasma antioxidant capacity of lipid-soluble compounds. 

The decreased plasma antioxidant capacity of water-soluble compounds by the seeds is associated with the decreased plasma uric acid (Figure 4C), which is strongly hydrophilic, but not with the plasma levels of albumin (Figure 4F) nor bilirubin (data not shown). All these components, together with vitamin C, are considered the main blood plasma antioxidants in humans [20,21]. Paradoxically, plasma uric acid positively correlates and predicts the development of obesity, hypertension, and cardiovascular disease [21]. Thus, the decreased plasma uric acid by the seeds in the present study can be considered beneficial for the body, especially when looking at its role in the development of gout. Support of this supposition can be found in the study by Opyd et al. [9], who showed that dietary supplementation with hemp seeds improved the antioxidant status of the liver in Zucker rats by increasing glutathione levels and decreasing a marker of lipid peroxidation. This means that a considerable decrease in the plasma antioxidant capacity and uric acid level does not automatically exclude benefits coming from hemp seed supplementation to the organ’s antioxidant defense system. 

Moreover, gamma-glutamyl transferase and blood plasma creatinine were neither modified by obesity itself (Figure 4E,H) nor by hemp supplementation, as opposed to blood plasma total protein level, which was decreased by HO. It is worth mentioning that obesity increased blood plasma albumins, total protein, and urea (Figure 4D,F,G,).

## 5. Conclusions

Dietary supplementation with ground hemp seeds was far more beneficial than with oil, which suggests that the lipid fractions, mainly including PUFAs, are only partially responsible for this effect. In both cases, dietary hemp supplementation was unable to attenuate the development of obesity with its complications, despite the cholesterol-lowering effect, some improvement in the vascular functioning, and changes in blood plasma antioxidant status.

## Figures and Tables

**Table 1:** The composition of hemp seeds and hemp seed oil ^1^.

	Hemp Seeds	Hemp Seed Oil
Dry matter (DM), %	93.1 ± 0.07	-
Crude fat, % DM	33.2 ± 0.36	-
Dietary fiber, % DM	27.5 ± 0.31	-
Crude protein, % DM	26.3 ± 0.49	-
Ash, % DM	5.09 ± 0.01	-
Nitrogen-free extract, % DM	1.05 ± 0.01	-
Fatty acid profile (%)		
Linoleic acid (18:2 n-6)	52.3 ± 0.07	52.8 ± 0.08
α-Linolenic acid (18:3 n-3)	18.1 ± 0.04	17.47 ± 0.03
Oleic acid (18:1 n-9)	7.91 ± 0.11	8.61 ± 0.05
Palmitic acid (16:0)	5.80 ± 0.01	5.46 ± 0.01
γ-Linolenic acid (18:3 n-6)	4.41 ± 0.02	4.26 ± 0.05
Stearic acid (18:0)	2.18 ± 0.01	2.26 ± 0.01
cis-11,14-Eicosadienoic acid (20:2 n-6)	1.61 ± 0.01	1.52 ± 0.01
Vaccenic acid (18:1 n-7)	0.95 ± 0.01	0.93 ± 0.02
Arachidic acid (20:0)	0.85 ± 0.00	0.83 ± 0.01
Gondoic acid (20:1 n-9)	0.41 ± 0.01	0.38 ± 0.01
Behenic acid (22:0)	0.36 ± 0.01	0.33 ± 0.01
Lignoceric acid (24:0)	0.15 ± 0.00	0.13 ± 0.01
cis-9,trans-12-Octadecadienoic acid (18:2 n-6)	0.11 ± 0.01	0.12 ± 0.00
Palmitoleic acid (16:1)	0.09 ± 0.00	0.08 ± 0.01
Calculated fatty acid content (%)		
PUFAs	76.4 ± 0.12	76.1 ± 0.03
n-3	18.1 ± 0.04	17.5 ± 0.02
n-6	58.3 ± 0.09	58.6 ± 0.02
MUFAs	9.37 ± 0.11	10.0 ± 0.03
SFAs	9.34 ± 0.02	9.02 ± 0.01
TFAs	0.11 ± 0.01	0.12 ± 0.00

^1^ Values are means ± SD (*n* = 3). DM, dry matter; MUFA, monounsaturated fatty acid; PUFA, polyunsaturated fatty acid; SFA, saturated fatty acid; TFA, trans fatty acid.

## Data Availability

Data supporting reported results can be found in Supplementary Materials.

## Supplementary Materials

The following are available online at https://www.mdpi.com/article/10.3390/nu13082575/s1, Table S1: Experimental results, Table S2: E_max_, pEC_50,_ and AUC values calculated from cumulative concentration–response curve from thoracic arteries of supplemented rats.
