# Supplementary material for: The Effect of Hemp (Cannabis sativa L.) Seeds and Hemp Seed Oil on Vascular Dysfunction in Obese Male Zucker Rats
Source: Nutrients. 2021 Jul 27;13(8):2575. doi: 10.3390/nu13082575 (PMC8398088; doi:10.3390/nu13082575)
Supplement: Supplementary file 1 [file nutrients-13-02575-s001.zip › nutrients-1296612-supplementary.pdf]

Table S1. Experimental results

Index	Mean				± SD			
	OC	O-HO	O-HS	LC	OC	O-HO	O-HS	LC
Initial body weight, g	224.9	222.6	217.7	176.3	12.88	21.45	18.74	12.40
Final body weight, g	370.2	367.7	349.9	267.0	27.45	19.88	25.15	13.85
Body weight gain, g	145.4	145.1	132.2	90.70	18.84	22.25	20.59	13.20
Dietary intake, g/day	23.89	23.40	23.60	15.15	2.238	1.424	1.176	0.620
TC, mmol/L	6.337	5.668	5.160	2.670	0.577	0.156	0.806	0.273
HDL-C, mmol/L	2.263	1.717	1.618	0.960	0.261	0.153	0.189	0.068
TG, mmol/L	5.305	2.940	4.011	2.347	1.674	1.405	0.912	0.821
log10(TG/HDL-C)	0.371	0.179	0.388	0.397	0.149	0.129	0.124	0.089
TC/HDL	2.809	3.321	3.199	2.783	0.168	0.278	0.440	0.224
TC minus HDL-C	4.073	3.952	3.542	1.710	0.380	0.180	0.724	0.237
VLDL-C (tg/2.2)	2.411	1.336	1.823	1.067	0.761	0.639	0.414	0.373
LDL-C	2.021	2.615	1.719	0.643	0.764	0.670	0.957	0.371
TG*TC*LDL-C/HDL-C	25.26	23.06	21.36	3.686	5.611	5.888	12.92	1.098
non-HDL-C/HDL-C	1.809	2.321	2.199	1.783	0.168	0.278	0.440	0.224
LDL-C/HDL-C	0.866	1.556	1.048	0.684	0.318	0.484	0.562	0.423
ACW, mg/mL	4.940	3.930	1.460	1.680	1.170	1.600	0.247	0.482
ACL, mg/mL	32.80	26.30	24.50	18.80	3.190	15.80	7.130	7.540
Uric acid, µmol/L	99.80	69.30	17.80	18.30	68.70	59.30	6.31	3.83
Urea, mmol/L	9.410	8.160	7.520	5.830	0.221	1.630	0.955	0.667
Creatinine µmol/L	6.450	4.320	9.570	10.30	3.390	2.930	4.850	4.500
Albumin, µmol/L	547.0	505.0	504.0	454.0	19.60	37.60	30.20	29.50
Total Protein, g/L	83.80	75.50	76.10	63.70	5.170	4.270	5.010	4.460
GGT, U/L	5.080	5.267	3.433	1.800	3.962	2.583	2.725	1.910
MDA in blood plasma, µmol/L	20.05	14.58	10.92	8.625	1.463	2.487	2.289	0.8419
MDA in the heart, ng/g	677.8	536.9	522.7	556.6	142.5	52.83	46.56	72.45

thromboxan A_2, pg/mL								
Basal	546.7	508.3	481.7	416.7	92.88	73.87	73.33	56.80
Stimulated with acetylcholine (10 μM)	873.3	840.0	806.7	658.3	81.65	74.50	72.57	73.60
Glycemia, mg/dL								
0	94.17	104.7	108.3	98.33	8.060	14.79	17.75	7.866
15	217.2	264.8	307.3	170.7	59.63	67.77	108.1	14.80
30	212.2	329.8	316.8	168.3	53.59	145.2	110.9	32.32
60	168.2	330.0	291.0	144.0	35.82	164.9	131.8	18.60
90	175.0	283.2	287.7	154.2	19.79	113.6	129.8	16.79
120	163.2	228.8	253.8	136.8	25.69	80.21	103.2	14.81
180	107.3	145.2	147.7	116.7	11.72	49.08	47.28	11.18

Table S2. Emax, pEC_{50} and AUC of thoracic arteries

	OC			O+HO			O+HS			LC		
	Top	$LogEC_{50}$	AUC	Top	$LogEC_{50}$	AUC	Top	$LogEC_{50}$	AUC	Top	$LogEC_{50}$	AUC
**Acetylcholine**	72.79	7.05	167.50	80.15	6.83	171.50	84.44	7.13	203.50	85.83	6.97	207.00
±	2.96	0.11	17.80	3.10	0.10	20.82	3.67	0.11	29.72	3.42	0.10	24.34
**Sodium nitroprusside**	103.70	8.90	437.20	107.50	8.51	377.00	118.70	8.42	399.50	96.00	9.58	422.50
±	1.48	0.05	10.64	4.24	0.14	11.82	4.02	0.14	16.81	2.97	0.09	19.61
**Pinacidil**	98.52	5.69	91.00	97.74	6.23	133.80	100.90	6.17	133.30	95.12	6.67	165.80
±	4.01	0.09	9.67	3.78	0.09	16.24	4.38	0.10	19.47	3.79	0.09	19.50
**NS1619**	14.01	5.73	3.50	110.10	7.65	243.60	98.84	10.75	288.00	93.09	6.07	110.50
±	0.20	0.03	0.09	4.34	0.13	7.64	3.34	0.18	12.12	2.88	0.06	5.13
**Noradrenaline**	124.00	6.69	230.50	194.80	6.92	366.50	181.10	7.01	356.00	163.10	7.01	313.00
±	5.05	0.10	23.00	7.54	0.10	25.00	7.86	0.11	34.00	6.50	0.10	26.00
**U-46619**	293.90	8.03	561.50	271.50	7.91	490.00	310.60	7.59	476.00	227.10	7.45	312.00
±	82.29	0.17	35.00	76.02	0.15	34.00	86.97	0.14	41.00	63.59	0.17	29.00
